# Perceived quality of collaboration in dehydration care among Dutch nursing home professionals: A cross‐sectional study

**DOI:** 10.1111/jan.15149

**Published:** 2022-01-03

**Authors:** Simone J. C. Paulis, Irma H. J. Everink, Ruud J. G. Halfens, Christa Lohrmann, Jos M. G. A. Schols

**Affiliations:** ^1^ Department of Health Services Research and Care and Public Health Research Institute (CAPHRI) Maastricht University Maastricht The Netherlands; ^2^ Institute of Nursing Science Medical University of Graz Graz Austria; ^3^ Department of Family Medicine and Care and Public Health Research Institute (CAPHRI) Maastricht University Maastricht The Netherlands

**Keywords:** care professionals, collaboration, dehydration, nurses, nursing home, questionnaire

## Abstract

**Aim:**

To explore the perceived quality of collaboration in dehydration care among nursing and medical staff in Dutch nursing homes.

**Design:**

A cross‐sectional study.

**Methods:**

An online questionnaire was administered to nursing and medical staff in February 2020 to assess the quality of collaboration in dehydration care and its influencing factors. Descriptive statistics, chi‐square tests and multinomial logistic regression analysis were used to describe the results and examine differences between groups.

**Results:**

In total, 695 questionnaires were completed by multiple levels of (specialized) nursing staff and nursing home physicians. The quality of collaboration was assessed as good (23.2%), sufficient (59.4%) and insufficient (17.4%). Predicting factors related to perceiving the quality of collaboration as good were working experience, dehydration training during education and the presence of a dehydration protocol/guideline in the nursing home. Enabling factors related to collaboration in dehydration care were ‘availability of sufficient aids to detect dehydration’, ‘continuity in the care relationship’ and ‘sufficient background data of the resident in the care record’. Factors that hinder collaboration were ‘insufficient knowledge about dehydration among nursing and medical staff’, ‘the absence of a team meeting in which the topic dehydration is discussed’ and ‘insufficient staffing level among nursing and medical staff’.

**Conclusion:**

Collaboration in dehydration care was generally assessed as sufficient. Participants with >10 years of working experience, who received dehydration training during their education and had a dehydration protocol/guideline available in the nursing home, perceived the quality of collaboration more often as good. Experienced barriers and enablers for collaboration in dehydration care varied between professional groups. Therefore, it is important to gain more insight into (informal) caregivers’ perceptions on what is expected from each other about dehydration care.

**Impact:**

Care professionals experience several limiting factors in collaborating in dehydration care. Addressing these factors could optimize dehydration care in Dutch nursing homes.

## 1 INTRODUCTION

Dehydration is a care problem that often occurs in nursing homes (Toles & Anderson, 2011). Not only do physiological changes due to ageing increase the dehydration risk, but also care dependency, multimorbidity and polypharmacy result in a higher risk of dehydration in nursing home residents (Wojszel, 2020). Dehydration does not always occur acutely (e.g. due to vomiting or infection) but may develop gradually and sometimes unnoticed because of insufficient fluid intake over a longer period of time. Dehydration can affect a resident's quality of life because of its associated negative health outcomes (e.g. renal failure, pressure ulcers and impaired cognitive function). Therefore, it is important to timely recognize signs and symptoms and prevent dehydration from happening (Mantantzis et al., 2020; Rodriguez et al., 2015). To achieve this, sufficient knowledge and expertise among care professionals in nursing homes to observe and diagnose signs and symptoms related to dehydration are needed. Moreover, multiple (groups of) professionals in the nursing home are involved in dehydration care and perform different activities (Bak et al., 2017). These activities vary from observing residents and regularly interacting with them to detect changes in clinical status (such as drinking less than normal or having diarrhoea), to taking action if dehydration is suspected (e.g. give the resident more fluid or start a fluid balance chart). Also communicating findings to colleagues or taking the decision to do a (blood) test to assess if dehydration is present, are important activities in dehydration care. Because these different activities are often performed by more than one professional, dehydration care is, therefore, a shared responsibility, and makes adequate collaboration a requisite (Paulis et al., 2021). This study examines how nursing and medical staff rate the quality of collaboration in dehydration care in their nursing home.

### 1.1 Background

Dutch nursing homes employ their own professionals, including nursing home physicians (NHPs – only present in the Netherlands), advanced nurse practitioners (ANPs), registered nurses (RNs), certified nurse assistants (CNAs), nurse assistants (NAs) and allied health professionals (Backhaus, 2017; Lovink et al., 2017; Rondas et al., 2015; Verenso, 2015). Next to these professionals, informal caregivers have a (voluntary) supportive role in Dutch nursing homes, for instance by providing drinks and food during their daily visits (Roberts & Ishler, 2018). This means that informal caregivers can also observe important changes in the health status of the resident. A study performed among CNAs and RNs in Dutch nursing homes reported that even though they know from theory which signs/symptoms are related to dehydration, they did often not observe these signs/symptoms in daily practice themselves. This was probably caused by overlapping activities across professionals in the nursing home, and the aforementioned supportive role informal caregivers have. Another conclusion of this study was that a clear description of roles about dehydration care in, and between, formal and informal caregivers was lacking and that it is unknown to what extent collaboration in dehydration care currently exists (Paulis et al., 2021). Collaboration can be defined as ‘a complex phenomenon that brings together two or more individuals, who work to achieve shared aims and objectives’ (Fewster Thuente & Velsor Friedrich, 2008). Research underlines that collaboration between professionals is essential in geriatric care because of the complexity of the health status and social circumstances of older adults and good collaboration may lead to more efficient and effective care (Young et al., 2011). However, achieving optimal collaboration in nursing homes is a challenge. Difficulties in adapting collaboration especially in nursing homes are, amongst others inadequate care documentation, a lack of interprofessional communication and a lack of competences (Mueller et al., 2014). As many (in)formal caregivers are present in Dutch nursing homes and information on how these caregivers collaborate in dehydration care is not available, it is important to examine their collaborating experiences and find out if this collaboration needs to be optimized to approach dehydration effectively.

## 2 THE STUDY

### 2.1 Aim

The aim of the study was to examine how nursing and medical staff rate the quality of collaboration in dehydration care in Dutch nursing homes.

### 2.2 Study design

A cross‐sectional study was conducted in February 2020, using an online questionnaire.

### 2.3 Participants

To be included in this study, participants had to meet the following inclusion criteria:

- Their profession was NHP, ANP, RN, CNA and NA.
- Participants were currently working in a Dutch nursing home.
- Participants gave consent for the use of their answers for scientific purposes.

Participants were recruited through three different channels: (1) the Dutch association for NHPs (Verenso, 2015) and the association for ANPs, RNs and CNAs (V&VN) (V&VN VS, 2021); (2) through educational institutes for ANPs, RNs, CNAs and NAs and (3) through Dutch nursing homes (convenience sampling). The first author send an e‐mail to the associations and institutes asking them to distribute the questionnaire among members, former students and employees. The e‐mail contained a separate invitation for the participants with information on the research purpose, general information on the questionnaire (e.g. time investment) and a questionnaire link to Qualtrics (University of Michigan‐Flint, 2021).

### 2.4 Data collection

#### 2.4.1 Online questionnaire

The questionnaire used in this study was part of a larger study and consisted of two parts. The first part of the questionnaire investigated which signs and symptoms of a diagnostic strategy to diagnose dehydration in the nursing home were associated with dehydration by nursing staff (Paulis et al., 2020). In addition, interventions taken by nursing staff after having observed factors associated with dehydration were examined (Paulis et al., 2021). The second part of the questionnaire examined the collaboration in dehydration care between multiple care professionals in Dutch nursing homes. The questionnaire for this larger study was developed by the research team (all authors) and is based on literature and expert opinion. This current article reports on the second part of this questionnaire, focused on collaboration. Besides reporting on collaboration, background characteristics of participants were taken into account. Participants were asked to indicate their profession (NHP, ANP, RN, CNA and NA), working experience (0–5 years, 5–10 years, 10–15 years, 15–20 years or >20 years), the nursing home population they mainly worked with (somatic, psychogeriatric or both), if they received dehydration training during their professional education (yes/no) and if they received dehydration training during their career (yes/no). Participants were also asked if a dehydration protocol/guideline was available in the nursing home they worked (yes/no). No minimum working experience requirement was set for participants to be included in this study because this study also wanted to include the amount of working experience as factor in the analyses.

In the questionnaire part ‘collaboration’, participants were asked to answer the following question:

How do you assess the current quality of the collaboration between nursing and medical staff about dehydration care in the nursing home you work (good, sufficient or insufficient)?

**FIGURE 1:**
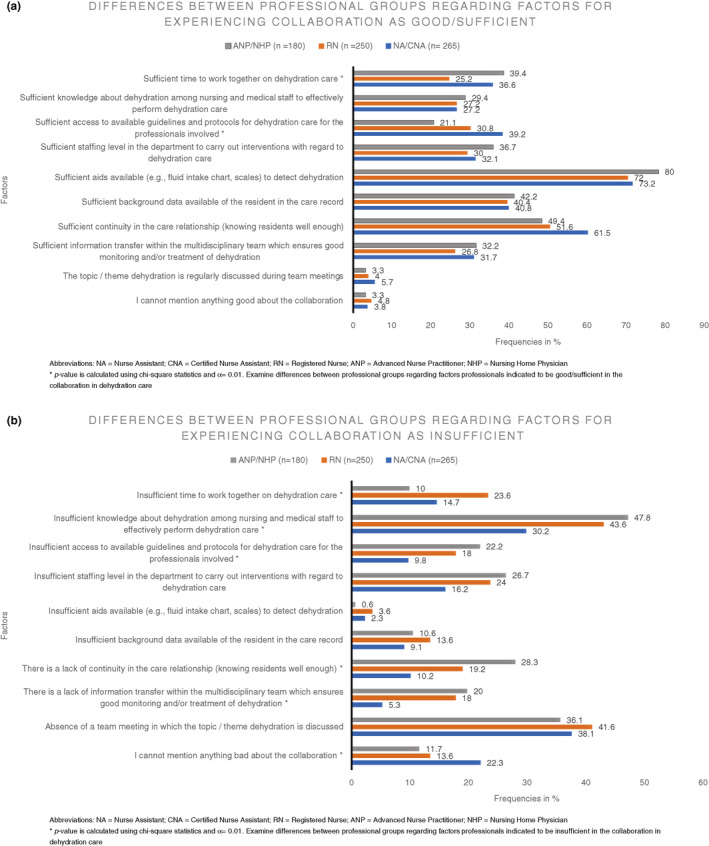
(a) Differences between professional groups about factors for experiencing collaboration as good/sufficient. (b) Differences between professional groups about factors for experiencing collaboration as insufficient]

Subsequently, to obtain more in‐depth information, participants were asked to substantiate their answer and indicate what they experienced as good/sufficient (e.g. sufficient time to work together on dehydration care) or insufficient (e.g. insufficient knowledge about dehydration among nursing and medical staff to effectively perform dehydration care) in the collaboration between nursing and medical staff about dehydration care (for all items see Figure 1a,b).

The questionnaire was assessed on content validity and clarity by a test panel (*n* = 12) consisting of NHPs (*n* = 3), ANPs (*n* = 2), RNs (*n* = 2), CNAs (*n* = 3) and NAs (*n* = 2), derived from national contacts from the corresponding author. The test panel was asked to verify if: (1) the purpose of the study was clear; (2) the questions matched the aim of the study; (3) the questionnaire was understandably worded and (4) any information was missing. After the test panel revised the questionnaire, textual adjustments were made, where after the questionnaire was finalized and entered into the software programme Qualtrics (University of Michigan‐Flint, 2019) (see Data S1).

### 2.5 Ethical considerations

The local Medical Ethics Committee of the University Hospital approved the study to be executed (2019‐1443). Participation was anonymous and on a voluntary basis. At the start of the questionnaire, participants had to agree with the use of their answers for scientific purposes. If participants did not agree, the questionnaire was closed.

### 2.6 Data analysis

Descriptive statistics in SPSS 26 IBM were used to analyse the answers given by the participants. To explore the relationship between the dependent variable ‘quality of collaboration’ (good, sufficient and insufficient) and independent variables ‘working experience’, ‘nursing home population’, ‘dehydration training (during education or during career)’, ‘dehydration protocol/guideline available in the nursing home’ and ‘professional group’, multiple chi‐square statistics was used. Variables that showed a significant relationship with ‘quality of collaboration’ (*p* ≤ .01) were entered into a multinomial logistic regression analysis. For the dependent variable (quality of collaboration), ‘good’ was considered as the reference group, while ‘sufficient’ and ‘insufficient’ were considered as comparative groups. The chi‐square statistics was applied to examine differences between professional groups about factors professionals indicated to be good/sufficient or insufficient in the collaboration in dehydration care. To gain comparable group sizes for data analysis, NAs and CNAs were combined into one professional group as well as ANPs and NHPs. The significance level used in this study was *α* = 0.01 (George & Mallery, 2019).

### 2.7 Validity, reliability and rigour

To guarantee the quality, the questionnaire used in this study was verified by a test panel for content and clarity. The test panel was conducted from national contacts from the first author. The test panel consisted of a representation of the participants used in this study namely: NAs (*n* = 2), CNAs (*n* = 3), RNs (*n* = 2), ANP (*n* = 2) and NHPs (*n* = 3). The questionnaire was distributed to the test panel by e‐mail or a printed version of the questionnaire was handed out. The test panel assessed whether (1) the questions of the questionnaire corresponded with the purpose of the study; (2) the wording of the questions was clear and understandable for the participants in this study and (3) if any information was missing. The test panel could send their feedback to the first author by writing or by e‐mail. Based on the feedback received from the test panel, the questionnaire was adjusted (textual changes) and finalized (see Data S1).

## 3 RESULTS

### 3.1 Characteristics and the quality of collaboration in dehydration care

In total, 695 participants completed the questionnaire consisting of three professional groups: 265 NAs/CNAs, 250 RNs and 180 ANPs/NHPs. The majority of NAs/CNAs often had >20 years of working experience (41.1%) compared with RNs (29.6%) and ANPs/NHPs (27.2%) who mostly had 0 to 5 years of working experience. Overall, the RNs (42.4%) and ANPs/NHPs (58.3%) worked with both somatic and psychogeriatric residents and a major part of the NAs/CNAs with psychogeriatric residents (52.1%). The majority of the participants received dehydration training during their education (78.4%) but not during their career (74.8%). More than half of the ANPs/NHPs did not have a dehydration protocol/guideline in the nursing home they worked (52.8%). Of the NAs/CNAs, 47.2% indicated to have a dehydration protocol/guideline in the nursing home they worked. RNs often did have a dehydration protocol/guideline present in their nursing home (37.6%) or did not know there was a dehydration protocol/guideline (37.6%). Lastly, all professional groups overall rated the quality of collaboration in dehydration care with sufficient (59.6%, 59.2% and 59.4%; see Table 1).

**TABLE 1:** Characteristics of participants and the perceived quality of collaboration in dehydration care

	NA/CNA (*n* = 265)^a^	RN (*n* = 250)	ANP/NHP (*n* = 180)^a^	Total (*n* = 695)
Working experience (years)				
0–5	23.0%	29.6%	27.2%	26.5%
5–10	11.7%	17.6%	26.7%	17.7%
10–15	14.0%	13.2%	11.1%	12.9%
15–20	10.2%	11.6%	11.1%	10.9%
>20	41.1%	28.0%	23.9%	31.9%
Nursing home population				
Somatic	20.0%	25.6%	15.6%	20.9%
Psychogeriatric	52.1%	32.0%	26.1%	38.1%
Both	27.9%	42.4%	58.3%	41.0%
Dehydration training during education				
Yes	70.6%	85.2%	80.6%	78.4%
No	29.4%	14.8%	19.4%	21.6%
Dehydration training during career				
Yes	24.9%	19.2%	33.9%	25.2%
No	75.1%	80.8%	66.1%	74.8%
Dehydration protocol/guideline available in the nursing home				
Yes	47.2%	37.6%	14.4%	35.3%
No	6.0%	24.8%	52.8%	24.9%
Don’t know	46.8%	37.6%	32.8%	39.9%
Quality collaboration				
Good	27.2%	20.4%	21.1%	23.2%
Sufficient	59.6%	59.2%	59.4%	59.4%
Insufficient	13.2%	20.4%	19.4%	17.4%

Abbreviations: ANP, Advanced Nurse Practitioner; CNA, Certified Nurse Assistant; NA, Nurse Assistant; NHP, Nursing Home Physician; RN, Registered Nurse.

^a^ NAs and CNAs are combined into one professional group as well as ANPs and NHPs.

Participants with >10 years of working experience more often rated the quality of collaboration with good (28.4%) or sufficient (58.5%), than with insufficient (13.1%; *p* ≤ .001). In addition, receiving dehydration training during their education was more often present among participants who rated the quality of collaboration with good (25.9%) or sufficient (59.1%), than with insufficient (15.0%; *p* ≤ .001). Receiving training during the participants’ career made participants overall rate the quality of collaboration as good (31.4%) or sufficient (57.7%) than with insufficient (10.9%; *p* = .002). The quality of collaboration was mostly indicated as good (35.1%) or sufficient (54.7%) than with insufficient (10.2%) when a dehydration protocol/guideline was present in the nursing home participants worked (*p* ≤ .001). No significant relations were found for ‘nursing home population’ and ‘professional group’ (see Table 2).

**TABLE 2:** Association of characteristics with perceived quality of collaboration

	Good (*n* = 161)^a^	Sufficient (*n* = 413)^a^	Insufficient (*n* = 121)^a^	*p*‐value
Working experience^b^				
<10 years	16.6%	60.6%	22.8%	<.001*
>10 years	28.4%	58.5%	13.1%	
Nursing home population				
Somatic	24.1%	65.5%	10.3%	.103
Psychogeriatric	22.3%	56.6%	21.1%	
Both	23.5%	58.9%	17.5%	
Dehydration training during education				
Yes	25.9%	59.1%	15.0%	<.001*
No	13.3%	60.7%	26.0%	
Dehydration training during career				
Yes	31.4%	57.7%	10.9%	.002*
No	20.4%	60.0%	19.6%	
Dehydration protocol/guideline available in the nursing home				
Yes	35.1%	54.7%	10.2%	<.001*
No	15.0%	60.1%	24.9%	
I don’t know	17.7%	63.2%	19.1%	
Professional group^c^				
NA/CNA	27.2%	59.6%	13.2%	.117
RN	20.4%	59.2%	20.4%	
ANP/NHP	21.1%	59.4%	19.4%	

Abbreviations: ANP, Advanced Nurse Practitioner; CNA, Certified Nurse Assistant; NA, Nurse Assistant; NHP, Nursing Home Physician; RN, Registered Nurse.

^a^ Perception of the quality of collaboration between nursing staff and medical staff about dehydration care.

^b^ Working experience answer categories ‘0–5’, ‘5–10’,’10–15’, ‘15–20’ and >20 years are combined into >10 and <10 years.

^c^ NAs and CNAs are combined into one professional group as well as ANPs and NHPs.

* *p*‐value is calculated using chi‐square statistics and *α* = 0.01. Compares the perception of quality of collaboration (good, sufficient and insufficient) about the characteristics working experience, nursing home population, dehydration training during education, dehydration training during career, the presence of a dehydration protocol/guideline in the nursing home and professional group.

Table 3 shows the results of the multinomial logistic regression analysis. The variables ‘working experience’, ‘dehydration training during education’ and ‘dehydration protocol/guideline available in the nursing home’ were significantly associated with ‘good’ quality of collaboration. When looking at Table 3, a ‘good’ quality of collaboration was more often experienced by participants with >10 years of working experience (*p* ≤ .001), by participants who received dehydration training during their education (*p* = .002) and by participants who had a dehydration protocol/guideline available in the nursing home (*p* = .001). This analysis showed that receiving training on dehydration during the career was not significantly associated with quality of collaboration (see Table 3).

**TABLE 3:** Determination of predictors of the participants’ perception of quality of collaboration

Predictor variables	Sufficient versus Good^a^			Insufficient versus Good^a^		
	*p*‐value	Odds ratio	95% CI	*p*‐value	Odds ratio	95% CI
Working experience (<10 years vs. >10 years)	0.017	1.629	1.090–2.436	<0.001*	2.599	1.545–4.371
Dehydration training during education (yes vs. no)	0.042	0.574	0.336–0.980	0.002*	0.364	0.194–0.682
Dehydration training during career (yes vs. no)	0.355	0.819	0.538–1.249	0.088	0.578	0.308–1.085
Dehydration protocol/guideline available in the nursing home (yes vs. don't know)	0.002*	0.500	0.326–0.768	0.001*	0.361	0.196–0.666
Dehydration protocol/guideline available in the nursing home (no vs. don't know)	0.454	1.230	0.716–2.113	0.055	1.876	0.986–3.569

Abbreviation: CI, confidence interval.

^a^ Participants’ experienced quality of collaboration in dehydration care.

* *p*‐value is calculated using multinomial logistic regression analysis and *α* = 0.01. Determines the predictors of the participants’ perception of quality of collaboration (good, sufficient or insufficient). ‘Good’ as perceived quality of collaboration was considered as the reference group.

### 3.2 Experiences on collaboration in dehydration care

The three most frequently mentioned factors which made participants experience the collaboration as good/sufficient were: (1) there are sufficient aids available (e.g. fluid intake chart and scales) to detect dehydration (74.5%); (2) there is sufficient continuity in the care relationship (knowing residents well enough) (54.8%) and (3) there is sufficient background data available of the resident in the care record (41.0%). Factors mentioned most frequently as reasons for insufficient collaboration were ‘there is insufficient knowledge about dehydration among nursing and medical staff to effectively perform dehydration care’ (39.6%), ‘there is no team meeting in which the topic/theme dehydration is discussed’ (38.8%) and ‘there is an insufficient staffing level in the department to carry out interventions with regard to dehydration care’ (21.7%).

When looking at differences between professional groups on which factors made them experience collaboration as good/sufficient, a significant difference was found for ‘sufficient time to work together on dehydration care’ (*p* = .003) which NAs/CNAs (36.6%) and ANPs/NHPs (39.4%) more often experienced as an enabler compared with RNs (25.2%). In addition, ‘sufficient access to available guidelines/protocols for dehydration care for the professionals involved’ (*p* ≤ .001) was more frequently indicated as an enabler for collaboration in dehydration care in the professional group NAs/CNAs (39.2%) than in RNs (30.8%) and ANPs/NHPs (21.1%). Taking the factors into account that made the professional groups experience collaboration as insufficient, ‘insufficient knowledge about dehydration among nursing and medical staff to effectively perform dehydration’ (*p* ≤ .001), ‘a lack of information transfer in the multidisciplinary team which ensures good monitoring and/or treatment of dehydration’ (*p* ≤ .001) and ‘a lack of the continuity in the care relationship (knowing residents well enough)’ (*p* ≤ .001) hinders RNs and ANPs/NHPs more frequently in the collaboration in dehydration care than it does for NAs/CNAs. Lastly, compared with RNs (13.6%) and ANPs/NHPs (11.7%), NAs/CNAs (22.3%) significantly more often indicated that they could not mention anything insufficient about the collaboration (*p* = .004; see Figure 1a,b).

## 4 DISCUSSION

The present study shows that 59.4% of the nursing and medical staff in Dutch nursing homes, rated the quality of collaboration in dehydration care as ‘sufficient’. Even though comparable research on (interdisciplinary) collaboration in dehydration care in the nursing home setting is rare, a study focused on the perceived quality of collaboration between nursing home professionals in palliative care in the Netherlands found relatively comparable results. In this study, participants gave a score for collaboration of 6.9–7.3 on a Likert scale from 1 to 10. This was perceived by authors as ‘relatively high’ (Khemai et al., 2020).

In our study, no significant differences were found in the assessed quality of collaboration between different professional groups (nurse assistants, nurses and medical staff) working in the nursing home. However, when looking at differences in background characteristics of the groups and comparing these to the quality of collaboration, some significant differences were found. First, ‘working experience’ was a predictor for the quality of collaboration. Having >10 years of working experience significantly more often resulted in assessing the quality of collaboration as ‘good’ than with ‘insufficient’ This finding is in line with literature suggesting that >10 years of working experience results in more effective communication skills, strengthening professionals collaboration performance (Rush et al., 2017). Also, receiving dehydration training during the participants’ education was a significant predictor for the quality of collaboration. Receiving dehydration training during education showed an increased likelihood for participants to experience the quality of collaboration with ‘good’ than with ‘insufficient’. As proven by Bjerrum et al. (2012) training not only increase knowledge and skills on a specific topic, it also changes behaviour and refreshes awareness of ones own roles and responsibilities. Therefore, it is expected that receiving more (interprofessional) training focused on dehydration in clinical practice might increase the quality of collaboration (Matziou et al., 2014). Moreover, the presence of a dehydration protocol/guideline in the nursing home was a significant predictor of the quality of collaboration in this study. Participants who had access to a dehydration protocol/guideline in the nursing home they worked, were more likely to experience the quality of collaboration as good. Literature supports the importance of this finding as the availability of a protocol/guideline provides evidence‐based and structured guidance to care professionals on how they have to act towards a health problem (Abrahamson et al., 2012). Therefore, the availability and use of a dehydration protocol/guideline should be motivated in nursing homes as it ensures an effective and consistent approach for multiple care professionals involved in dehydration care.

Even though no significant differences were found in perceived quality of collaboration between different professional groups, results imply as if both RNs and ANPs/NHPs were able to mention more factors for insufficient quality of collaboration. First, NAs/CNAs significantly more often indicated not being able to mention anything bad about the collaboration, compared with RNs and ANPs/NHPs. Also, RNs and ANPs/NHPs mentioned to experience inadequate transfer of information about the resident in the multidisciplinary team significantly more often compared with NAs/CNAs. A reason for this could be the difference in educational background: ‘collaborative learning’ is an important topic in the education of nurses and physicians. During their education, they learn to develop knowledge and skills on how to effectively collaborate as care professionals (Iqbal et al., 2016; Zhang & Cui, 2018). However, when looking at the content of educational curricula of NAs/CNAs, collaborative learning is hardly integrated (Hegner et al., 2009). Therefore, it could be that RNs and ANPs/NHPs are better educated on what good collaboration constitutes and therefore, have different expectations about the quality of this collaboration.

When looking at other factors explaining the assessed quality of collaboration, ‘a lack of continuity in the care relationship (knowing residents well enough)’ was less often mentioned as a barrier for collaboration in dehydration care for NAs/CNAs (21.4%) compared with RNs (38.1%) and ANPs/NHPs (40.5%). These might be explained by differences in roles between the groups: NAs/CNAs have a central role in providing basic care to residents, and interact with the resident on a daily basis. As a consequence, they know the resident very well. This enables them to notice changes in the clinical status of the resident, including signs/symptoms of dehydration (Holloway & McConigley, 2008). On the contrary, in Dutch nursing homes, RNs and ANPs/NHPs are mostly involved in acute and post‐acute care, and/or have more coordinating (RNs) and/or consulting roles (ANPs/NHPs). This means they have limited (structural) contact moments with residents (Backhaus, 2017). As a consequence, it is expected that RNs and ANPs/NHPs depend on the NAs/CNAs to notice signs and symptoms of dehydration in a resident (Holloway & McConigley, 2008).

Apparently, many RNs and ANPs/NHPs assess a lack of information transfer in the multidisciplinary team as a barrier for good dehydration care, questioning the quality and quantity of information transfer between all professionals involved. A reason for this could be that not only the professional groups included in this study have a central role in providing information on changes in the residents’ status, but that also allied health professionals and the informal caregiver play a large role (Paulis et al., 2021). Other research also claims that inefficient communication between care professionals, older adults and informal caregivers could be an important barrier in collaboration (Verwijs et al., 2020). Hence, more clarity is needed in the division of roles of formal and informal caregivers about dehydration care in the nursing home to guarantee sufficient information transfer.

### 4.1 Strengths and limitations

To the best of our knowledge, there is no research available about experienced quality of collaboration in dehydration care by care professionals in nursing homes. Therefore, the findings of this study are a first contribution in this field. Yet, some limitations should be addressed. First, a validated questionnaire was not available. Therefore, we developed a questionnaire ourselves. Even though this questionnaire was assessed among a panel of experts and adjusted accordingly, it was not validated, which might result in bias. Also, in this study, a large group of participants was reached (*n* = 695). As the questionnaire was anonymized, we do not know if the group is representative for the whole country or only specific areas. In this study, participants were asked to rate the quality of collaboration (good, sufficient or insufficient) and explain their answer by indicating which factors enabled or impeded this collaboration. However, in the questionnaire, the categories ‘good’ and ‘sufficient’ were taken together. As a consequence, we cannot distinguish between the difference in enabling and impeding factors about the categories ‘good’ and ‘sufficient’. Even though this resulted in a little less detail in our results, we believe we still have a complete picture of enabling and impeding factors about the quality of collaboration.

Lastly, this study investigated collaboration without discussing how professionals see their own role and responsibilities in this topic. Also, informal caregivers have an important role in dehydration care but have not been included in this study. Therefore, a recommendation for future research may be to also look at individual roles of both formal and informal caregivers to optimize dehydration care in Dutch nursing homes.

## 5 CONCLUSIONS

Overall, the quality of collaboration in dehydration care in nursing homes was assessed by nursing staff and medical staff as sufficient. Yet, professionals mentioned various factors which limit collaboration in dehydration care. Examples are a lack of continuity in the care relationship (knowing residents well enough) or a lack of information transfer in the multidisciplinary team to ensure good monitoring and/or treatment of dehydration. These results indicate there is still room for improvement. Results also showed that NAs/CNAs experienced fewer barriers in the collaboration in dehydration care compared with RNs and ANPs/NHPs. This may be explained by differences in their educational background about (interdisciplinary) collaboration. Moreover, an association between perceived quality of collaboration and the characteristics working experience, received dehydration training during the participants’ education and the presence of a dehydration protocol/guideline in the nursing home was found, stressing the importance of more (interprofessional) training focused on dehydration in clinical practice and the integrated use of a dehydration protocol/guideline. Lastly, more clarity is needed in roles and responsibilities of both formal and informal caregivers about dehydration care in the nursing homes.

## CONFLICT OF INTEREST

All authors declare that there has been no conflict of interest.

## AUTHOR CONTRIBUTIONS

All authors were qualified for authorship and met the following criteria: (1) all authors have made substantial contributions to conception and design, acquisition of data and analysis and interpretation of data; (2) all authors have been involved in drafting the manuscript or revising it critically for important intellectual content; (3) all authors have given final approval of the version to be published. Each author has participated sufficiently in the work to take public responsibility for appropriate portions of the content and (4) all authors agreed to be accountable for all aspects of the work in ensuring that questions related to the accuracy or integrity of any part of the work are appropriately investigated and resolved.

### PEER REVIEW

The peer review history for this article is available at https://publons.com/publon/10.1111/jan.15149.

## Supporting information

Supplementary Material

## Data Availability

Data available on request from the authors.
