# Supplementary material for: Perceived quality of collaboration in dehydration care among Dutch nursing home professionals: A cross‐sectional study
Source: J Adv Nurs. 2022 Jan 3;78(8):2357–66. doi: 10.1111/jan.15149 (PMC9545722; doi:10.1111/jan.15149)
Supplement: Supplementary file 1 — Supplementary Material [file JAN-78-2357-s001.doc]

**Detecting dehydration, how do we do that?**

- **Can we use your answers (anonymously) for scientific research?**
- Yes
- No
- **Where do you work?**
- In the nursing home
- Other

**GENERAL QUESTIONS**

1. **What is your profession?**

- Nurse assistant
- Certified nurse assistant
- Registered nurse
- Advanced nurse practitioner
- Nursing home physician

1. **How many years of working experience in the nursing home you have?**

- 0-5 years
- 5-10 years
- 10-15 years
- 15-20 years
- >20 years

1. **Do you (mainly) work with somatic or psychogeriatric patients?**

- (mainly) somatic patients
- (mainly) psychogeriatric patients
- both

1. **Did you receive training on dehydration during your education?**

- Yes
- No

1. **Did you receive training on dehydration during your career?**

- Yes
- No

1. **Is there a protocol/guideline for diagnosing and treating dehydration in the nursing home you work at?**

- Yes
- No
- I don`t know

**COLLABORATION**

Some additional questions about the care for residents with (a risk of) dehydration

**Identifying and treating dehydration requires good coordination between nursing staff (nurse assistants, certified nurse assistants and registered nurses) and medical staff (advanced nurse practitioners and nursing home physicians). How do you assess the current quality of the collaboration between nursing staff and medical staff regarding dehydration care in the nursing home you work?**

- Good
- Sufficient
- Insufficient

**Could you motivate your answer? (more answers are possible)**

**What I find good/sufficient about the collaboration is:**

- There is sufficient time to work together on dehydration care
- There is sufficient knowledge about dehydration among nursing and medical staff to effectively perform dehydration care
- There is sufficient access to available guidelines and protocols for dehydration care for the professionals involved
- There is a sufficient staffing level in the department to carry out interventions with regard to dehydration care
- There are sufficient aids available (e.g., fluid intake chart, scales) to detect dehydration
- There is sufficient background data available of the resident in the care record
- There is sufficient continuity in the care relationship (knowing residents well enough)
- There is a sufficient information transfer within the multidisciplinary team which ensures good monitoring and/or treatment of dehydration
- The topic / theme dehydration is regularly discussed during team meetings
- Other ….. (fill in)
- I cannot mention anything good about the collaboration

**What I find insufficient about the collaboration is:**

- There is insufficient time to work together on dehydration care
- There is insufficient knowledge about dehydration among nursing and medical staff to effectively perform dehydration care
- There is insufficient access to available guidelines and protocols for dehydration care for the professionals involved
- There is an insufficient staffing level in the department to carry out interventions with regard to dehydration care
- There are insufficient aids available (e.g., fluid intake chart, scales) to detect dehydration
- There is insufficient background data available of the resident in t care record
- There is a lack of continuity in the care relationship (not knowing residents well enough)
- There is a lack of information transfer within the multidisciplinary team which causes insufficient monitoring and/or treatment of dehydration
- Absence of a team meeting in which the topic / theme dehydration is discussed
- Other ….. (fill in)
- I cannot mention anything bad about the collaboration

**Last Question: Can we approach you for participation in follow-up research? If yes, please write down your e-mail address**

- Yes ………….
- No

**Thank you for your participation!**

**Click further to save your answers!**
